# Apremilast combined with low-dose corticosteroids for lichenoid drug eruption following programmed cell death protein 1 inhibitor and lenvatinib therapy: A case report

**DOI:** 10.1016/j.jdcr.2026.03.058

**Published:** 2026-04-08

**Authors:** Xiaoqi Meng, Dansheng Li, Bingrong Zhou, Juan Liu, Yang Xu

**Affiliations:** a Department of Dermatology, The First Affiliated Hospital with Nanjing Medical University, Nanjing, Jiangsu Province, China; b Department of Dermatology, Beilun District People’s Hospital, Ningbo, Zhejiang Province, China

**Keywords:** apremilast, lenvatinib, lichenoid drug eruptions, PD-1 inhibitor

## Introduction

Programmed cell death protein 1 (PD-1) inhibitors combined with the multitarget tyrosine kinase inhibitor (TKI) lenvatinib have become a first-line treatment strategy for various malignancies and have demonstrated favorable safety profiles in prior reports, with only rare cases of drug eruption following combination therapy. Current first-line treatment for drug eruption typically involves full-dose corticosteroids. However, reports have indicated that in patients with a history of malignancy, while corticosteroids may alleviate cutaneous lesions, their use could be associated with the risk of rapid tumor progression.

In recent years, the phosphodiesterase-4 (PDE-4) inhibitor apremilast has garnered increasing attention due to its favorable safety profile and demonstrated therapeutic efficacy. In the oncologic setting, PDE-4 inhibitors may be coadministered with low-dose corticosteroids to potentiate therapeutic efficacy while mitigating adverse outcomes.

Here we report a case of cutaneous lichenoid drug eruptions (LDEs) that developed in a patient with multiple primary malignancies receiving combination therapy with the PD-1 inhibitor sintilimab and lenvatinib. The eruption markedly improved after treatment with the PDE-4 inhibitor apremilast without evidence of tumor progression.

## Case report

A 56-year-old man (66 kg) with a history of multiple primary malignancies—including prostatic adenocarcinoma, renal clear cell carcinoma, and moderately to poorly differentiated hepatocellular carcinoma—had previously undergone radical prostatectomy, left nephrectomy, and partial hepatectomy. Postoperatively, he received combination therapy with the sintilimab (200 mg every 3 weeks) and the lenvatinib (12 mg/day). Two weeks after initiation of therapy, scattered erythematous papules appeared on the trunk and extremities, accompanied by severe pruritus. The eruption progressively worsened over a 10-month period, eventually evolving into a generalized rash. Despite discontinuation of both agents for more than 1 month, the lesions persisted. During this period, oral loratadine and topical halomethanes were administered, with minimal improvement.

**Fig 1:**
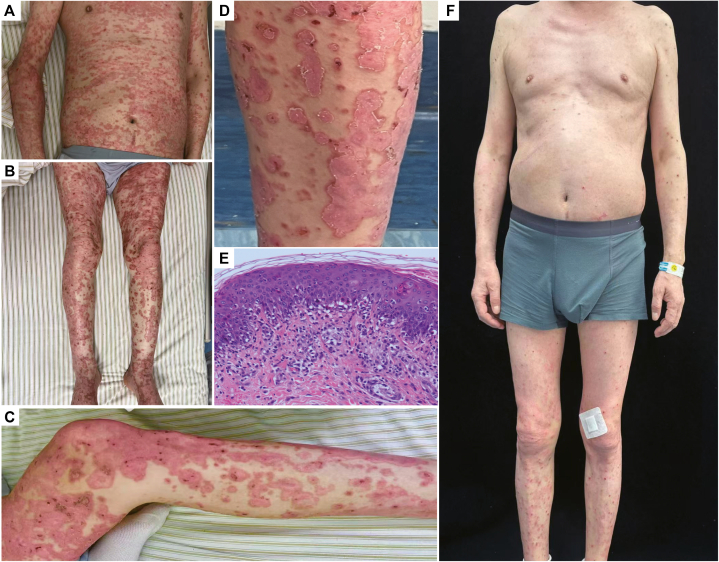
Clinical and histopathological features of dermatitis medicamentosa following combined lenvatinib and PD-1 inhibitor therapy. **A**-**D,** Multiple targetoid erythematous lesions coalescing into map-like patterns with scaling and crusting. **E,** H&E histology showing parakeratosis, dyskeratotic keratinocytes, basal cell vacuolar degeneration, and mild-to-moderate perivascular lymphoeosinophilic infiltrates in the superficial to mid-dermis. **F,** Marked improvement after 4 weeks of low-dose corticosteroids followed by apremilast. *H&E*, Hematoxylin and eosin; *PD-1*, programmed cell death protein 1.

**Fig 2:**
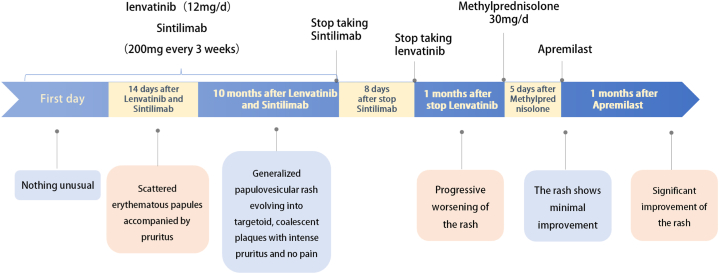
Clinical timeline of disease onset, treatment, and recovery.

Physical examination revealed annular erythematous plaques with central inflammatory papules and vesicles, partially coalescing into map-like configurations (Fig 1, *A*-*D*). The lesions were intensely pruritic. Histopathologic examination demonstrated mild epidermal hyperplasia, parakeratosis, numerous dyskeratotic keratinocytes, focal inflammatory cell exocytosis, and widespread basal vacuolar degeneration with pigment incontinence. The superficial and mid-dermis exhibited perivascular inflammatory infiltrates composed predominantly of lymphocytes, histiocytes, and eosinophils (Fig 1, *E*). Direct immunofluorescence revealed no immunoglobulin or complement deposition at the dermo-epidermal junction. Serological testing for autoantibodies associated with autoimmune blistering diseases was negative, as were tests for antinuclear antibodies, extractable nuclear antigen antibodies, and anti-Ro/anti-La antibodies. These clinical and histopathologic findings supported a diagnosis of drug-induced dermatitis secondary to combination therapy with a PD-1 inhibitor and lenvatinib. Given the patient’s history of multiple malignancies, a short course of low-dose systemic methylprednisolone therapy (prednisone 30 mg/day) was initiated, followed by titrated with the PDE4 inhibitor apremilast (30 mg twice daily). Over the subsequent 2 weeks, methylprednisolone was successfully tapered and discontinued. Marked clinical improvement was observed within 4 weeks following administration of apremilast, which was initiated while the patient was off both cancer-directed agents (Fig 1, *F*). The clinical timeline is shown in Fig 2.

## Discussion

Lenvatinib, an oral TKI, selectively targets vascular endothelial growth factor receptors 1-3, fibroblast growth factor receptors 1-4, KIT, RET, and platelet-derived growth factor receptor-α.^1^ It has been approved by the U.S. Food and Drug Administration for the treatment of thyroid carcinoma, renal cell carcinoma, hepatocellular carcinoma, and endometrial carcinoma. In contrast to most other TKIs, lenvatinib uniquely inhibits fibroblast growth factor receptor-mediated signaling, which plays a critical role in multiple cellular cascades, including cell proliferation, differentiation, growth, and apoptosis. This broader inhibitory profile may underlie both its antitumor efficacy and its distinct spectrum of adverse events. Cutaneous toxicities associated with lenvatinib have been increasingly recognized in clinical practice, with reported manifestations such as palmar-plantar hypoesthesia, psoriasiform eruptions, and pyoderma gangrenosum. The pathophysiological mechanisms responsible for these dermatologic events remain incompletely understood. It has been proposed that inhibition of vascular endothelial growth factor receptor signaling may induce keratinocyte apoptosis or disrupt endothelial–stromal interactions, leading to epidermal barrier dysfunction and inflammation.^2^ Moreover, lenvatinib exerts a pronounced immunomodulatory effect. Previous studies have demonstrated an increased proportion of circulating T lymphocytes in patients receiving lenvatinib compared with controls.^1^ Cutaneous adverse events associated with anti-PD-1 immune checkpoint inhibitors are relatively common.^3^ The underlying mechanisms include PD-1 blockade–induced T-cell activation, which promotes an inflammatory state, and loss of T-cell homeostasis, leading to self-directed cytotoxic responses.^4^ LDE is an uncommon but increasingly recognized adverse cutaneous reaction. It typically presents as lichenoid skin lesions, predominantly involving the extensor surfaces of the extremities or manifesting with generalized distribution. Recent literature implicates anti-TNF-α monoclonal antibodies, TKIs, and checkpoint inhibitors as its most common triggers.^5^

Apremilast is an oral small-molecule PDE-4 inhibitor that elevates intracellular cAMP levels, activates cAMP-dependent signaling pathways, and modulates proinflammatory cytokines such as TNF-α, thereby exerting anti-inflammatory effects. Approved by the U.S. Food and Drug Administration for psoriasis and psoriatic arthritis, it has also shown favorable efficacy and safety in off-label use for various inflammatory and immune-mediated dermatoses.^6^ However, its role in drug-induced dermatitis (including LDE) and lichen planus has not yet been reported.

Only 2 cases of Stevens–Johnson syndrome/toxic epidermal necrolysis associated with combined PD-1 inhibitor and lenvatinib therapy have been documented, both requiring prolonged high-dose corticosteroids; 1 succumbed to the condition, while the other exhibited rapid tumor progression.^7^^,^^8^ Given the patient’s history of multiple malignancies, a short course of low-dose glucocorticoids followed by apremilast achieved satisfactory efficacy without evidence of tumor progression. It is noteworthy that the resolution of the rash with apremilast took place in the absence of continued cancer therapy, suggesting a direct therapeutic effect of apremilast rather than simply recovery from drug withdrawal.

To our knowledge, this is the first reported case of an LDE induced by sintilimab plus lenvatinib, and the first to demonstrate successful treatment with apremilast, without subsequent elevation of tumor. These findings highlight the need for careful monitoring for cutaneous eruptions during combined lenvatinib and PD-1 inhibitor therapy and indicate that apremilast combined with low-dose corticosteroids is a safe and effective intervention for managing LDE in cancer patients, with the distinct advantage of facilitating corticosteroid tapering and eventual discontinuation.

## Conflicts of interest

None disclosed.
